# A novel minimally invasive percutaneous treatment for Essex-Lopresti joint depression-type DIACFs by ligamentotaxis

**DOI:** 10.1186/s12893-022-01868-6

**Published:** 2022-12-16

**Authors:** Zhiguo Chen, Chongyin Fan, Jinsong Zhang, Chen Zhao, Xin Du, Wei Huang, Weidong Ni, Gang Luo

**Affiliations:** Department of Orthopedics, Orthopedic Laboratory of Chongqing Medical University, The First Affiliated Hospital of Chongqing Medical University, 1 Youyi Rd, Chongqing, 400016 China

**Keywords:** Calcaneus, Intra-articular fracture, Minimally invasive, Percutaneous fixation

## Abstract

**Objective:**

To compare the clinical efficacy of minimally invasive percutaneous treatment by ligamentotaxis with traditional open reduction and internal fixation in the treatment of Essex-Lopresti joint depression-type displaced intra-articular calcaneal fractures (DIACFs).

**Methods:**

The medical records of patients with calcaneal fractures admitted to our department from January 2016 to December 2020 were retrospectively analyzed, and patients who met the inclusion criteria were finally included for analysis. Twenty-one patients underwent minimally invasive percutaneous treatment by ligamentotaxis (Group A), while eighteen patients were treated by traditional open reduction and internal fixation through an extended lateral approach (Group B). The preoperative waiting time, operative time, hospital stay, radiologic parameters (calcaneal height, width, length, Böhler angle and Gissane angle), American Foot and Ankle Surgery Association (AOFAS) hindfoot scores, Maryland Foot Score (MFS), visual analogue scale (VAS), and incidence of complications of the included patients were all recorded and analysed.

**Results:**

Thirty-nine patients with Essex-Lopresti joint depression type DIACFs were finally included. According to the Sanders classification, 22 were type II, 12 were type III and 5 were type IV. The preoperative waiting time and the hospital stay of Group A were 3.7 ± 1.6 d and 7.2 ± 1.7 d, respectively, which were significantly shorter than those of Group B (6.9 ± 2.0 d and 12.4 ± 1.5 d) (P < 0.05). There was no significant difference in the operative time between the two groups (88.8 ± 9.8 min vs. 91.3 ± 12.1 min; P > 0.05). No significant differences were shown in the radiological parameters (calcaneal height, width, length, Böhler angle and Gissane angle) or the satisfactory rate of joint surface reduction (SRJSR) of the two groups immediately postoperatively. All patients were followed up for 14 to 56 months [(30.2 ± 10.4) months]. All fractures healed. At the final follow-up, there were no significant differences in the radiological parameters or the SRJSR between the two groups (P > 0.05). No significant differences were shown in the AOFAS scores, MFS or VAS scores between the two groups [(89.5 ± 8.2) vs. (89.4 ± 9.0), P > 0.05; (87.5 ± 8.3) vs. (86.3 ± 8.9), P > 0.05; and (2.1 ± 1.2) vs. (2.2 ± 1.2), P > 0.05]. The excellent and good rates of the AOFAS scores and MFS were 90.5% and 85.7%, respectively, in Group A and 88.9% and 88.9%, respectively, in Group B (P > 0.05). Four patients experienced wound complications, including 1 superficial incision infection, 2 skin necrosis around the incision edge and 1 deep infection in Group B, while there were no wound complications in Group A (P < 0.05). One patient in each group suffered traumatic arthritis (P > 0.05).

**Conclusions:**

In the assessment of Essex-Lopresti joint depression type DIACFs, minimally invasive percutaneous treatment by ligamentotaxis has similar clinical outcomes to traditional open reduction and internal fixation through an extended lateral approach. However, the former has the advantages of shorter preoperative waiting time and hospital stay, and lower incidence of incision complications.

## Background

Intra-articular calcaneal fractures comprise approximately 75% of the calcaneal fractures. Displaced intra-articular calcaneal fractures (DIACFs) are the most difficult to treat. An assessment of functional status (SF-36) in patients with DIACFs showed that the functional prognosis of DIACFs was much worse than that of other calcaneal fractures [1], which indicates that patients with DIACFs have a potential risk of delayed or nonreturn to work. Therefore, reasonable management is of tremendous importance to DIACF patients. Compared with nonoperative treatments, a reasonable surgery can improve the functional prognosis of patients with DIACFs [2, 3]. Open reduction and internal fixation (ORIF) through an extended lateral approach has been the most commonly used technology for the treatment of DIACFs in the last 30 years [4–7]. This technology can clearly expose the operative field to reconstruct the integrity of the articular surface under direct vision, and it is especially suitable for the treatment of complex calcaneal fractures [5, 8]. However, it is indisputable that this technology tends to result in a higher incidence of wound complications [2, 9–11].

In view of this, some scholars began to use percutaneous minimally invasive technology to treat DIACFs, including closed reduction by ligamentotaxis and percutaneous screw fixation, and this technology did reduce the incidence of wound complications [12–14]. However, this technology has high operative requirements and requires good percutaneous reduction and fixation technology; otherwise, poor reduction and loss of reduction may occur [8, 15]. Essex-Lopresti percutaneous prying reduction [16] is the most widely used percutaneous reduction technology at present. For tongue-type calcaneal fractures, this technology can achieve good reduction [17, 18]. However, for joint depression-type calcaneal fractures, it is still difficult to use this technology for prying reduction, and poor reduction of the posterior articular surface may result from insufficient reduction [15, 19]. Hence, we introduced the percutaneous prying and jacking reduction technique into the treatment of DIACFs and combined it with the ligamentotaxis technique and critical positions fixation technique to form a novel minimally invasive percutaneous treatment for DIACFs.

In the present study, we compared the clinical efficacy of minimally invasive percutaneous treatment by ligamentotaxis with traditional ORIF in the treatment of Essex-Lopresti joint depression-type DIACFs. The purposes of the present study were as follows: (1) to explore the efficacy and advantages of minimally invasive percutaneous treatment by ligamentotaxis for Essex-Lopresti joint depression type DIACFs; (2) to summarize the surgical technique of this technology; and (3) to summarize the complications of this technology.

## Method

The study was authorized by the Medical Ethics Committee of the First Affiliated Hospital of Chongqing Medical University (Chongqing, China) (Ethical No. 2020-414) and was performed according to the ethical standards of the Declaration of Helsinki of 1964. Written informed consent was signed by all patients included.

### Study design and patients

The medical records of patients with calcaneal fractures who were admitted to our hospital from January 2016 to December 2020 were retrospectively reviewed.

Inclusion criteria: (1) age ≥ 16 years; (2) unilateral Essex-Lopresti joint depression type DIACFs; (3) fresh and closed fracture (within 3 weeks); (4) underwent percutaneous reduction and screw fixation or open reduction and internal fixation through an extended lateral approach; and (5) follow-up data were complete.

Exclusion criteria: (1) multiple injuries; (2) severe osteoporosis; (3) severe medical diseases (severe renal insufficiency, hyperthyroidism, stroke or myocardial infarction in nearly 3 months); and (4) unbearable surgery.

### Preoperative management

In order to assess the morphological changes of the calcaneus, the degree of joint surface collapse and to inform Sanders and Essex-Lopresti classification, lateral, anteroposterior, axial X-ray, CT and two-dimensional reconstruction of the calcaneus were performed. Usually, for the purpose of detumescence, the affected limb was elevated and applied ice after injury. In Group B, we need to wait for skin "wrinkle signs" before surgery, while in Group A it was not necessary.

### Surgical techniques

**Fig. 1:**
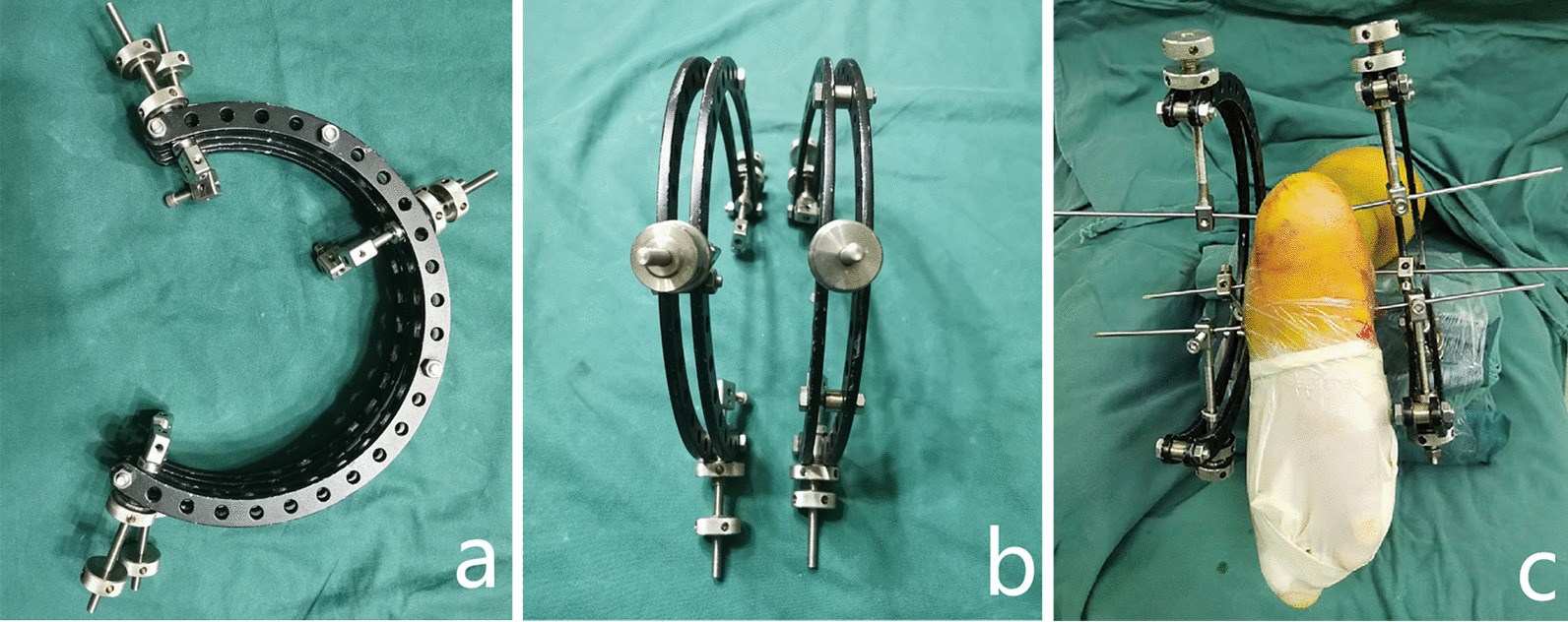
Homemade TCDR (Chinese Patent No.: 20192254154.4) (cited from Gang et al. [20]) (**a**,** b**). Clinical photograph showed homemade TCDR was set up on both side of the calcaneal (**c**)

**Fig. 2:**
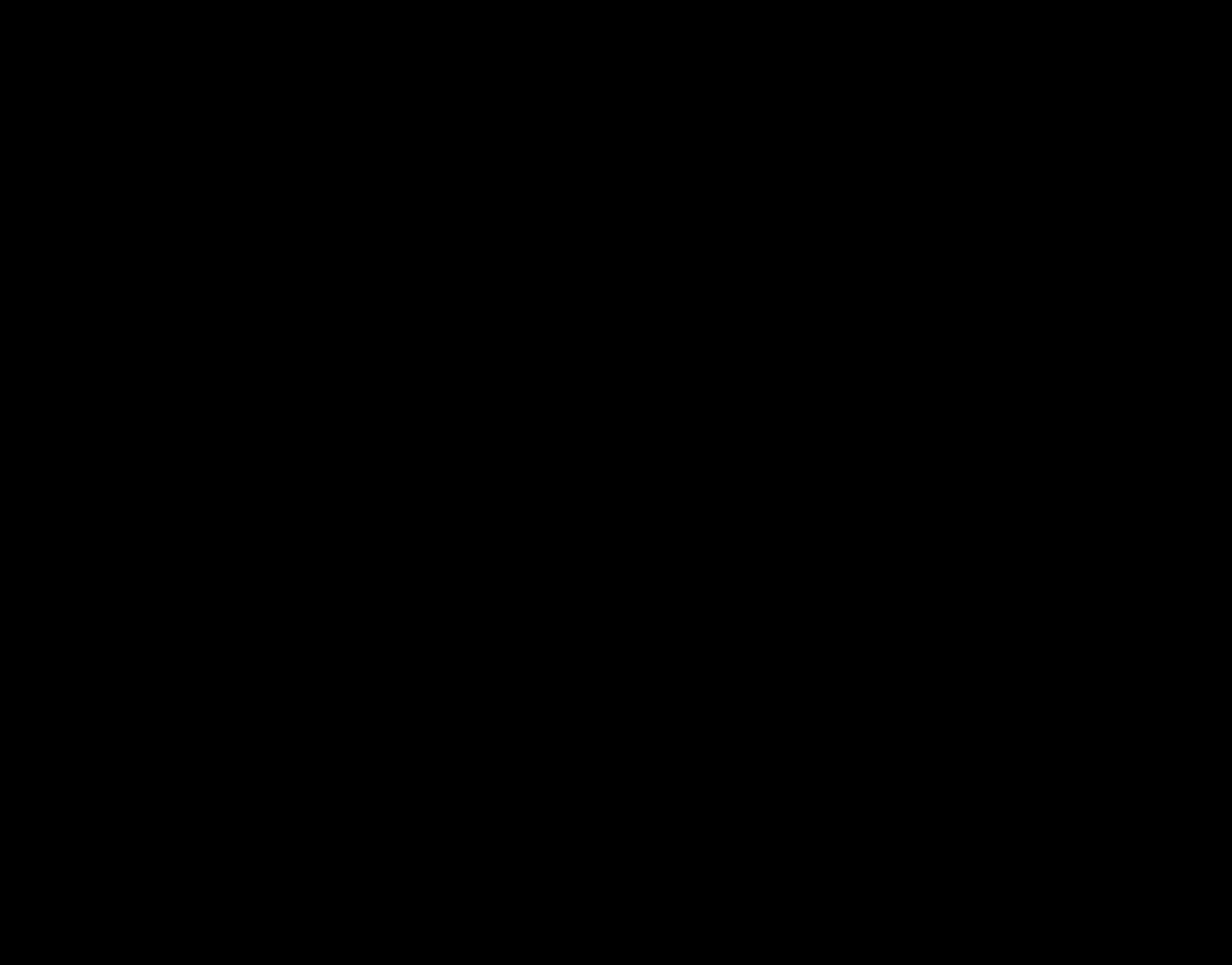
Percutaneous prying and jacking reduction technique. A 2.0 mm K-wire was drilled into the compressed fracture fragment under lateral and axial fluoroscopic guidance (**a**, **b**), then a hollow punch was inserted along the k-wire to reduce the compressed fracture fragment (**c**,** d**). After reduction, the guided K-wire was drilled through the compressed fracture fragment to the talus to temporarily fix the compressed fracture fragment (**e**, **f**) (Picture a and c cited from Gang et al. [20])

**Fig. 3:**
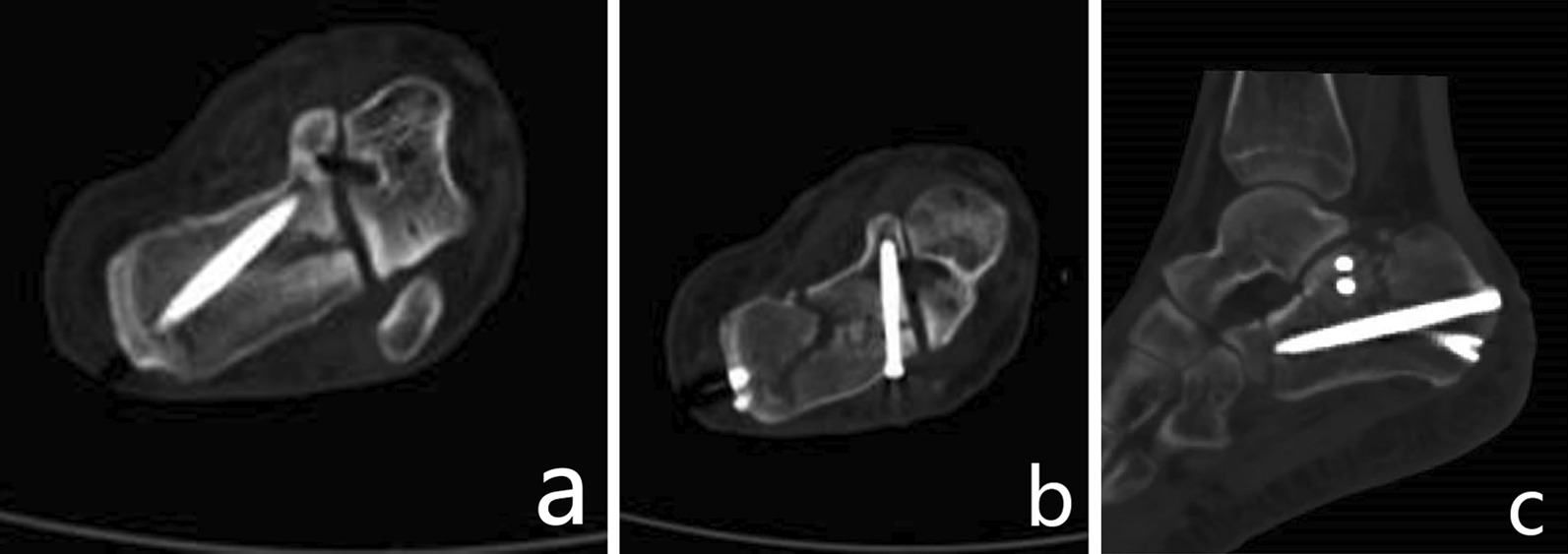
The specific orientation of the screws. The first screw was from calcaneal tuberosity to sustentaculum tali (**a**), the second screw was from calcaneal thalamus to sustentaculum tali (**b**), the third screw was from calcaneus tuberosity to calcaneus anterior tubercle (**c**) (Picture a and b cited from Gang et al. [20])

The surgical procedures of percutaneous reduction and screw fixation were as follows. After general or epidural anesthesia, the patient was placed in the prone position with routine antibiotic prophylaxis before the operation. The main surgical steps were as follows. (1) Using a homemade tri-plane calcaneal distraction reductor (TCDR) (Chinese Patent No. 201922254154.4) to restore the morphology (height, length and varus/valgus angulation) of the calcaneus (Fig. 1). The detailed process was showed in our previous article [20]. (2) Using the percutaneous prying and jacking reduction technique (Fig. 2) to reduce the articular surface fracture fragments. A 2.0 mm K-wire was drilled into the compressed fracture fragment under lateral and axial fluoroscopic guidance to ensure that it was in the best position. Then, a hollow punch was inserted along the k-wire to reduce the compressed fracture fragment. (3) After confirmation with C-arm fluoroscopy, a 5.5 mm all-cortical cannulated screw was placed along the calcaneal axis to maintain the height of the calcaneum (Fig. 3a). Then, one or two 3.5 mm or 4.3 mm cannulated screws were placed to fix the articular surface fracture fragments (Fig. 3b). Finally, another 5.5 mm all-cortical cannulated screw was placed along the long axis of the calcaneus to maintain the calcaneal length (Fig. 3c). The detailed process was showed in our previous article [20]. The wound was then closed through full-thickness suture.

The surgical procedures of ORIF were as follows. The patient was placed in a lateral decubitus position on the uninjured side and received spinal or general anaesthesia with routine antibiotic prophylaxis prior to the operation. An extended lateral approach and an L-shaped incision were made. The subperiosteal flap was made by sharp dissection to protect the surrounding soft tissue. After the fracture line was clearly visible, the broken lateral wall fragment was raised from the calcaneus as a covering. This clearly reveals the collapsed posterior articular surface. The induction and fixation steps were as follows. (1) To restore the morphology of the calcaneum, a Schanz screw or Steinmann pin was used to tract the calcaneal tubercle for the purpose of restoring the height and length of the calcaneus and correcting the varus/valgus alignment of the calcaneum. Then, a K-wire was drilled along the medial wall to temporarily maintain the morphology of the calcaneum. If traction reduction was difficult, a bone stripper could be inserted into the medial wall of the calcaneus for auxiliary reduction. (2) To reduce and fix the articular surface fracture fragment, the articular surface fracture fragment was reduced under direct vision, and then a K-wire or cannulated screw was used to maintain the reduction. (3) To fix the plate, the plate was placed after fluoroscopy confirmed that the height and length of the calcaneum were restored, the varus/valgus alignment was corrected, and the reduction of the articular surface was satisfactory. A negative pressure drainage tube was routinely placed in the incision before the incision was closed layer by layer.

### Postoperative management

Nonweight-bearing exercises, including active and passive flexion and extension of the toes and ankles, were performed immediately after the operation. Two weeks postoperatively, Inversion and eversion of the ankle and subtalar joint were performed, and circle exercises were started 1 month after the operation. The weight-bearing depended on the fracture healing. Generally speaking, full weight-bearing and supported squat exercises were started 8–12 weeks postoperatively. Outpatient review was generally conducted at 1, 3, 6 and 12 months after the operation, once a year after one year postoperatively.

### Clinical evaluation

The American Orthopaedic Foot and Ankle Society (AOFAS) hindfoot scores and Maryland Foot Score (MFS) were used to assess the functional outcomes. The AOFAS ankle-hindfoot scale consists of subjective and objective variables, and these variables were classified into three major categories (pain, function and alignment). And so is the MFS. The total scores for both scales range from 0 to 100, with higher score indicating better function. For this assessment, these two scales were both divided into four categories: 90 to 100 points were rated excellent; 75 to 89 points were good; 50 to 74 points were fair; less than 50 points were poor. Visual analogue scale (VAS) was used to assesse the pain with a range of 0–10, with 0 indicating the best outcome and 10 indicating the worst outcome. Postoperative complications included incisions and other related complications. Traumatic arthritis was evaluated comprehensively by the clinical symptoms and imaging findings.

### Radiological evaluation

Immediately after surgery, lateral, axial X-ray and CT scans were performed to evaluate the reduction of the morphology and posterior articular surface. At every follow-up evaluation, lateral and axial X-ray of the injured foot were performed to evaluate the maintenance of reduction. At the final follow-up, the anatomical parameters of the following were measured with radiographs, including the calcaneal height, width, length, Böhler angle and Gissane angle.

### Statistical analysis

Analyses were performed using SPSS statistical software version 21.0 software (SPSS Inc., Chicago, IL, USA). The quantitative parameters were described as the means and standard deviations (SDs), and the qualitative parameters were expressed as frequencies or percentages. Continuous variables were analysed through independent t tests, while qualitative variables were analysed through chi-squared tests. All hypotheses were evaluated with 2-sided tests with statistical significance set at a p value < 0.05.

## Results

**Fig. 4:**
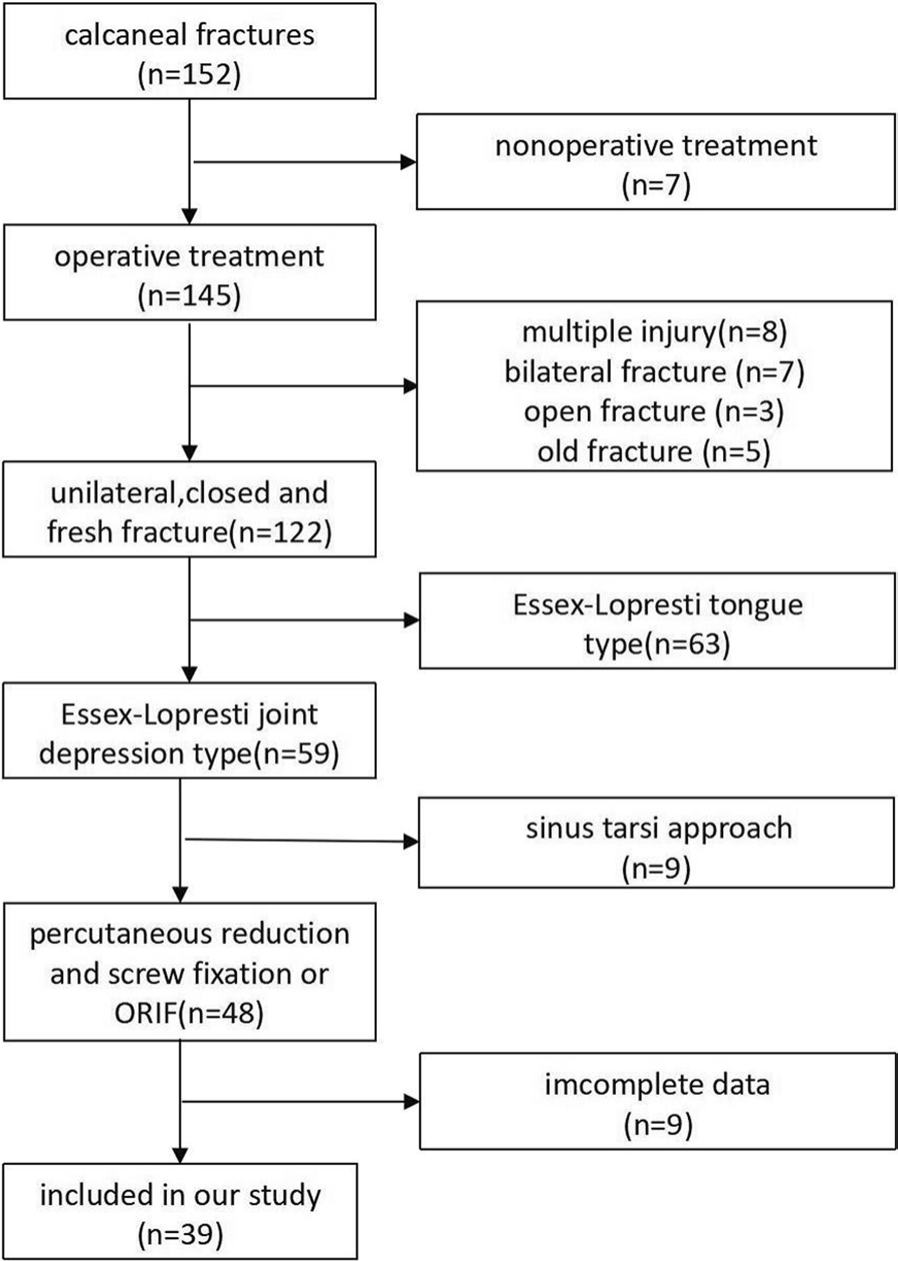
Patient selection flow chart

Finally, a total of 39 patients including 32 males and 7 females aged 26 to 61 years [47.1 ± 9.2 years] were enrolled in this study (Fig. 4). They are all joint compression type according to the Essex-Lopresti classification, and 22 were type II, 12 were type III and 5 were type IV according to the Sanders classification. There are thirty-four fall injuries and 5 traffic injuries (Table 1). The preoperative waiting time and the hospital stay of Group A were 3.7 ± 1.6 d and 7.2 ± 1.7 d, respectively, which were significantly shorter than those of Group B (6.9 ± 2.0 d and 12.4 ± 1.5 d) (P < 0.05). There was no significant difference in the operative time between the two groups (88.8 ± 9.8 min vs. 91.3 ± 12.1 min, P > 0.05) (Table 1).

**Table 1:** Comparison of the general characteristics of the two groups

	Group A (n = 21)	Group B (n = 18)	χ^2^/*t* value	*p* value
Sex (M/F)	17/4	15/3	0.037^a^	> 0.05
Age (years), (x ± s)	47.5 ± 8.5	46.6 ± 10.0	0.156^b^	> 0.05
Side of injured (R/L)	10/8	11/10	0.039^a^	> 0.05
BMI	22.3 ± 2.0	22.2 ± 2.1	0.096^a^	> 0.05
Smoking	5	4	0.140^b^	> 0.05
Comorbidity				
Diabetes	2	1	0.215^b^	> 0.05
Venous insufficiency	0	1	1.197^b^	> 0.05
Injure mechanism				
Falling accident	18	12	0.087^a^	> 0.05
Traffic accident	3	6		
Sanders classification				
Type II	12	10	0.487^a^	> 0.05
Type III	7	5		
Type IV	2	3		
Preoperative waiting time (days)	3.7 ± 1.6	6.9 ± 2.0	− 5.56^b^	< 0.05
Operation time (mins)	88.8 ± 9.8	91.3 ± 12.1	− 0.72^b^	> 0.05
Hospital stay (days)	7.2 ± 1.7	12.4 ± 1.5	− 9.87^b^	< 0.05

BMI: body mass index; SD: standard deviation

^a^Chi-squared test

^b^Independent-samples *t*-tests

The follow-up periods ranged from 14 to 56 months (30.2 ± 10.4 months). All fractures healed. There were no patients unable to walk normally due to shoe-wearing limitations. All patients returned to their preinjury working and living conditions after the operation in Group A, while in Group B, one patient chose to retire after the operation due to being near retirement. At the final follow-up, no significant differences were shown in the AOFAS scores, MFS or VAS scores between the two groups [(89.5 ± 8.2) vs. (89.4 ± 9.0), P > 0.05; (87.5 ± 8.3) vs. (86.3 ± 8.9), P > 0.05; and (2.1 ± 1.2) vs. (2.2 ± 1.2), P > 0.05] (Table 2). According to the AOFAS scores, 9 cases were excellent, 10 were good and 2 were fair, with an excellent/good rate of 90.5% in Group A, while 7 cases were excellent, 9 were good and 2 were fair, with an excellent/good rate of 88.9% in Group B (χ^2^ = 9.00, p > 0.05). According to MFS, 8 cases were excellent, 10 were good and 3 were fair, with an excellent/good rate of 85.7% in Group A, while 7 cases were excellent, 9 were good, and 2 were fair, with an excellent/good rate of 88.9% in Group B (χ^2^ = 12.00, p > 0.05).

**Table 2:** The function parameters between the two groups at final follow-up

	Group A (n = 21)	Group B (n = 18)	*t* value	*p* value
AOFAS	89.5 ± 8.2	89.4 ± 9.0	0.03	> 0.05
MFS	87.5 ± 8.3	86.3 ± 8.9	0.43	> 0.05
VAS	2.1 ± 1.2	2.2 ± 1.2	− 0.20	> 0.05

AOFAS: American Foot and Ankle Surgery Association; MFS: Maryland Foot Score; VAS: visual analogue scale

No significant differences were shown in the radiological parameters (calcaneal height, width, length, Böhler angle and Gissane angle) or the satisfactory rate of joint surface reduction (SRJSR) of the two groups immediately postoperatively (P > 0.05) (Table 3). At the final follow-up, there were no significant differences in the radiological parameters between the two groups (P > 0.05) (Table 4). No significant differences were shown between the immediate postoperative and final follow-up radiological parameters of either group (P > 0.05) (Tables 5, 6), which indicated that there was no reduction lost in these two groups at the final follow-up.

**Table 3:** The immediately post-operative radiographic parameters between the two groups

	Group A (n = 21)	Group B (n = 18)	χ^2^/*t* value	*p* value
Calcaneal height (mm)	39.2 ± 1.9	39.7 ± 2.4	− 0.70^a^	> 0.05
Calcaneal length (mm)	71.9 ± 2.4	71.1 ± 1.9	1.21^a^	> 0.05
Calcaneal width (mm)	33.9 ± 2.2	32.8 ± 1.3	1.84^a^	> 0.05
Böhler angle (°)	28.3 ± 4.7	28.4 ± 2.7	− 0.08^a^	> 0.05
Gissane angle (°)	125.8 ± 5.1	126.5 ± 6.1	− 0.386^a^	> 0.05
SRJSR	1/21	1/18	0.01^b^	> 0.05

SRJSR: satisfactory rate of joint surface reduction

^a^Chi-squared test

^b^Independent-samples *t*-tests

**Table 4:** The radiographic parameters between the two groups at final follow-up

	Group A (n = 21)	Group B (n = 18)	*t* value	*p* value
Calcaneal height (mm)	39.0 ± 1.9	39.6 ± 2.4	− 0.74	> 0.05
Calcaneal length (mm)	71.8 ± 2.3	71.3 ± 1.6	1.21	> 0.05
Calcaneal width (mm)	34.0 ± 1.9	33.1 ± 1.2	1.60	> 0.05
Böhler angle (°)	28.4 ± 4.5	28.2 ± 2.5	0.17	> 0.05
Gissane angle (°)	125.9 ± 5.0	126.6 ± 5.9	− 0.43	> 0.05

**Table 5:** Comparison of the radiographic parameters between immediate post-operative and final follow-up in group A

	Immediate post-operative	Final follow-up	*t* value	*p* Value
Calcaneal height (mm)	39.2 ± 1.9	39.0 ± 1.9	0.68	> 0.05
Calcaneal length (mm)	71.9 ± 2.4	71.8 ± 2.3	1.00	> 0.05
Calcaneal width (mm)	33.9 ± 2.2	34.0 ± 1.9	− 0.57	> 0.05
Böhler angle (°)	28.3 ± 4.7	28.4 ± 4.5	− 0.90	> 0.05
Gissane angle (°)	125.8 ± 5.1	125.9 ± 5.0	− 0.37	> 0.05

**Table 6:** Comparison of the radiographic parameters between immediate post-operative and final follow-up in group B

	Immediate post-operative	Final follow-up	*t* value	*p* value
Calcaneal height (mm)	39.7 ± 2.4	39.6 ± 2.4	0.40	> 0.05
Calcaneal length (mm)	71.1 ± 1.9	71.3 ± 1.6	− 1.46	> 0.05
Calcaneal width (mm)	32.8 ± 1.3	33.1 ± 1.2	− 1.20	> 0.05
Böhler angle (°)	28.4 ± 2.7	28.2 ± 2.5	0.26	> 0.05
Gissane angle (°)	126.5 ± 6.1	126.6 ± 5.9	− 1.00	> 0.05

The incidence of wound complications in Group A was significantly lower than that in Group B (0.0% vs. 22.2%, χ^2^ = 5.20, p < 0.05). No wound complications were found in Group A. While 4 cases suffered wound complications in Group B, 1 case had superficial infection of the incision and was cured after anti-infection treatment. Two cases had skin necrosis around the incision, of which one case was epidermal necrosis, which was cured after dressing change, and the other case was full-thickness flap necrosis, which was cured after debridement and suture. One case of deep infection was cured after anti-infection treatment, debridement, antibiotic bone cement packing and VSD covering. One case of traumatic arthritis occurred in both Groups A and B (χ^2^ = 0.01, p > 0.05), which manifested as pain in the tarsal sinus area when walking on an uneven road. However, oral administration of NSAIDs significantly relieved the symptoms, and no surgical intervention was required for the two patients.

**Fig. 5:**
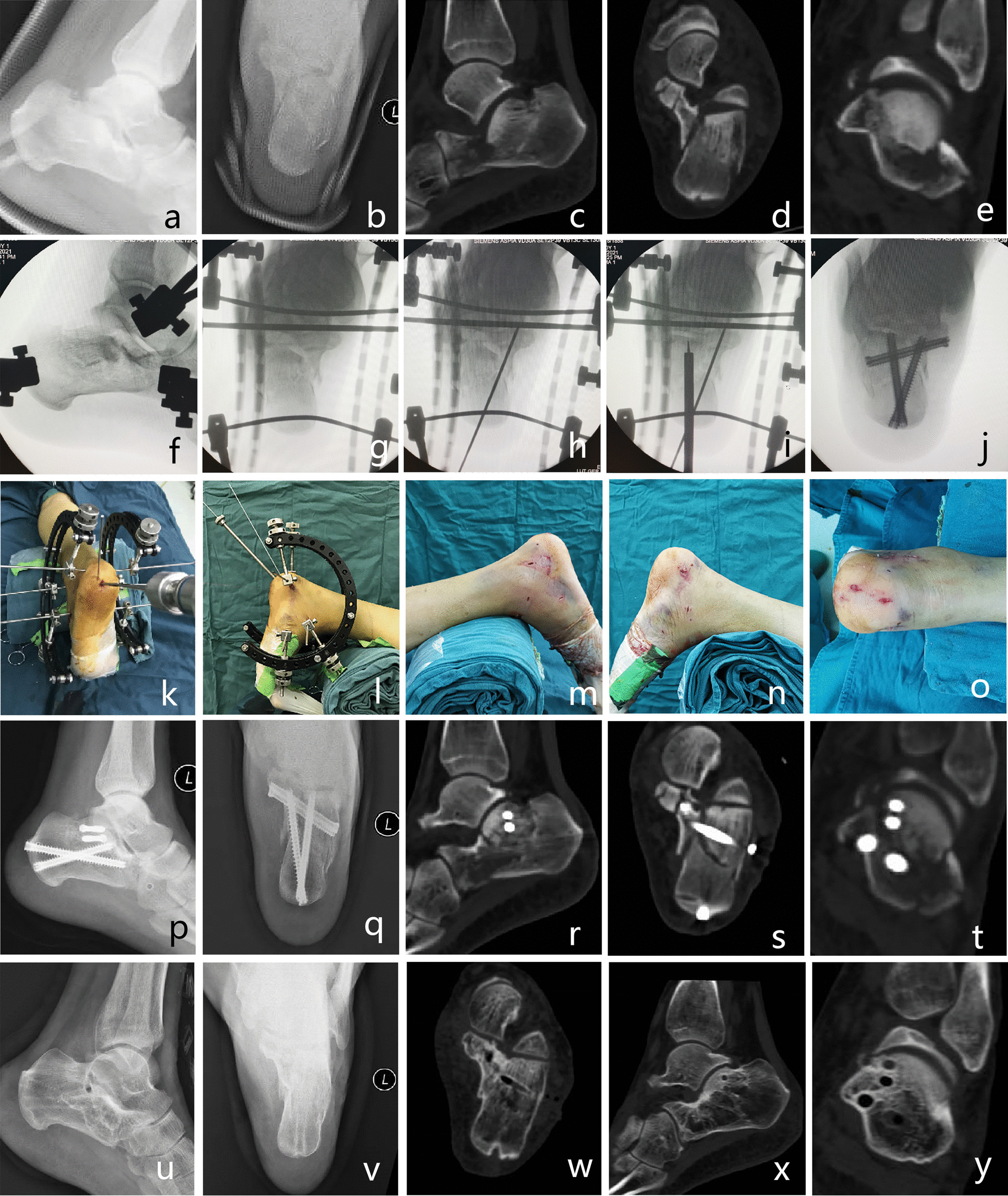
A 32-year-old male patient suffered left displaced intra-articular calcaneal fracture caused by a fall from a height, Essex-Lopresti classification (joint compression type), Sanders classification (II C). Preoperative lateral (**a**) and axial (**b**) X-rays, axial (**c**), sagittal (**d**) and coronal (**e**) CT views showed significantly decreased calcaneal height, Bohler angle and Gissanes angle, significantly increased calcaneal width and significantly collapse of the subtalar articular surface. Intraoperative fluoroscopy (**f–j**) showed the surgical procedures: (1) restored the morphological of the calcaneus by the TCDR (**f**, **g**), (2) temporarily fixed the medial calcaneus column with K-wire (**h**), (3) reduced the articular surface fracture fragments (**i**)and (4) percutaneously fixed by cannulated screws (**j**). Clinical photograph showed the percutaneous prying and jacking reduction technique (**k**,** l**) and the surgical incision (**m–o**). Immediate postoperative lateral (**p**) and axial (**q**) X-rays, axial (**r**), sagittal (**s**) and coronal (**t**) CT views showed ideal reduction of the calcaneal height/width, subtalar articular surface, Bohler angle and Gissanes angle. 14 months postoperative lateral (**u**) and axial (**v**) X-rays, axial (**w**), sagittal (**x**) and coronal (**y**) CT views showed fracture has healed without reduction lose

Figure 5 shows the typical case.

## Discussion

### Efficacy and advantages

The minimally invasive percutaneous treatment for DIACFs by ligamentotaxis can achieve ideal reduction and durable stable fixation. In the present study, immediately after the operation, there were no significant differences in radiologic parameters and a satisfactory rate of joint surface reduction between the two groups, indicating that this technology could achieve the same reduction quality as the classical operation. At the last follow-up, there was no joint surface subsidence and reduction lost or screw malposition, indicating that percutaneous minimally invasive screw fixation had perfect biomechanical stability. However, it is worth noting that further analysis of the radiologic parameters revealed a 12.2% increase in the width of the calcaneus compared to the normal, but fortunately, none of the patients had lateral malleolar impingement signs or shoe-wearing limitations at the final follow-up. In fact, effectively restoring and maintaining the calcaneal width is a difficult problem in the treatment of calcaneal fractures with percutaneous screw fixation. Tomesen et al. [14] used percutaneous reduction and screw fixation to treat 39 patients with DIACFs, of which Sanders type IV accounted for 33.3%. Although the overall excellent and good rate was close to 80%, the calcaneal width was widened by approximately 23.6% compared with the normal, resulting in limited shoe wearing in some patients. We think that there are two main reasons for the dissatisfaction with the correction of calcaneal widening when using percutaneous minimally invasive technology to treat DIACFs: (1) dissatisfaction with the reduction of the collapsed articular surface fracture block was not conducive to the recovery of the calcaneal width, which was the main reason and could be confirmed in a linked paper by Tomesen et al. [14]; and (2) for patients with severe comminution of the lateral wall, even the calcaneal width was restored satisfactorily, conventional screw fixation could not effectively maintain the calcaneal width. Therefore, for patients with severe comminution of the lateral wall, the choice of screw fixation should be made with caution.

The advantages of minimally invasive percutaneous treatment by ligamentotaxis for Essex-Lopresti joint depression type DIACFs were as follows. (1) The incidence of wound complications was significantly reduced. In the present study, the wound complication rate in the minimally invasive group was much lower than that in the ORIF group, which was consistent with that reported in related papers [12–14]. (2) The preoperative waiting time and hospital stay were shortened. As DIACFs are often accompanied by serious soft tissue injury, for ORIF, we have to waiting for "wrinkle signs" for the purpose of reducing the incidence of wound complications [4]. However, for percutaneous minimally invasive treatmen, there was no necessary waiting for the appearance of "wrinkle signs", so the preoperative waiting time was shortened than that of ORIF.

### Reduction techniques

**Fig. 6:**
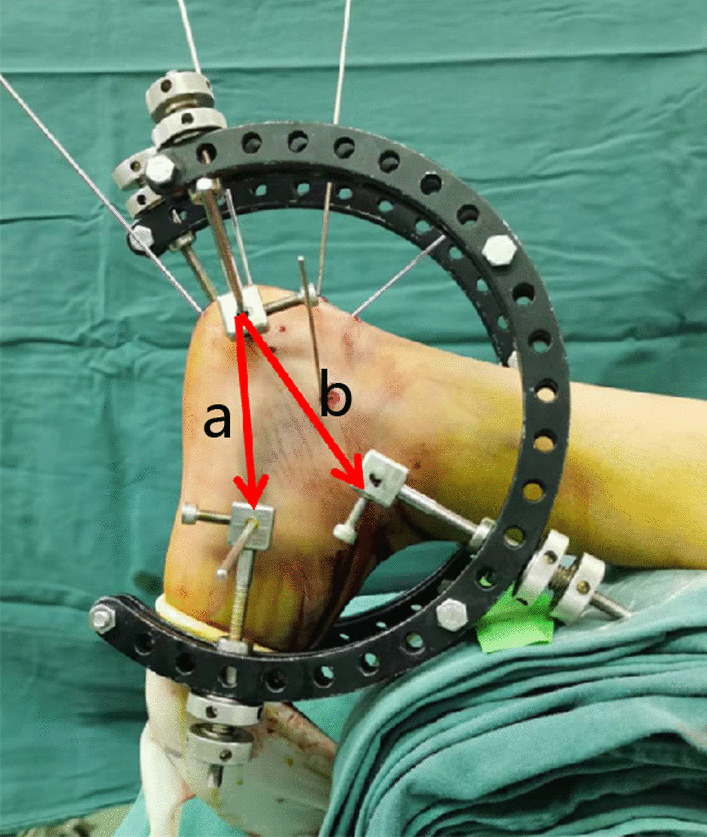
The orientations of the tensions between the three K-wires. The tension between calcaneal tuberosity and cuboid bone is parallel to the calcaneal length axis (**a**), the tension between calcaneal tuberosity and talus neck is parallel to the height axis of the calcaneum (**b**)

The reduction of Essex-Lopresti joint depression-type DIACFs includes recovery of the calcaneal morphology (length, width, height, varus and valgus) and reduction of the collapsed articular fracture fragment. In this study, we used ligamentotaxis to restore the morphology of the calcaneum with a homemade tri-plane calcaneal distraction reductor (TCDR) (Chinese Patent No. 201922254154.4). The reduction techniques were as follows. (1) The three traction points of TCDR were located in the calcaneal tuberosity, talus neck and cuboid bone. The tension formed between the calcaneal tuberosity and talus neck was parallel to the height axis of the calcaneum, conducive to the recovery of the calcaneal height. The tension between the calcaneal tuberosity and cuboid bone was parallel to the calcaneal length axis, which was conducive to the recovery of the calcaneal length (Fig. 6). (2)When the calcaneum alignment was varus, the medial distraction force should be greater than that of lateral, which is conducive to correcte varus alignment. In contrast, the lateral distraction force should be greater than that of medial.

For the collapsed articular fracture fragment, we used prying and jacking techniques for reduction (Fig. 2). The reduction techniques included the following. (1) The reduction process was carried out in the traction maintenance state, as a widen subtalar joint space was conducive for reduction of the collapsed fragment. (2) The guided K-wire was drilled under the guidance of fluoroscopy to ensure it was in the proper position. Usually, the k-wire was inclined to the side with more serious collapse, which was conducive to reduction. (3) The K-wire was used as a guide pin, and then a hollow punch was inserted along the k-wire to reduce the compressed fracture fragment. (4) The guided K-wire was directly used as a fixed K-wire to decrease redundant operations and avoid the loss of reduction. That is, when fluoroscopy indicated the reduction of the compressed fracture fragment was satisfactory, the guided K-wire was drilled through the compressed fracture fragment to the talus to maintain the reduction. (5) After the reduction of the compressed fracture fragment, the lateral wall of the calcaneus was squeezed to restore the width of the calcaneus.

### Fixation techniques

The most common complications of percutaneous screw fixation are screw withdrawal and reduction loss [15], which were closely related to the seriousness of the fracture comminution, the selection of the screw fixation position and screw orientation. To reduce these complications, we summarized the percutaneous key-point fixation principle of the core fracture blocks (Fig. 3). The fixation techniques were as follows. (1) The position of the screw fixation: three key-point positions needed to be fixed, namely, the calcaneal tuberosity, calcaneal thalamus and anterior calcaneal process. (2) The orientation of the screws: the first screw (screw A) was drilled into the sustentaculum tali from the inner side of the calcaneal tuberosity along the longitudinal axis of the calcaneus (Fig. 3a). The cortex of the sustentaculum tali was hard and formed a strong medial load-bearing column with the inner wall of the calcaneus, so this screw could well maintain the height of the calcaneum. The second screw (screw B) was drilled from the calcaneal thalamus to the sustentaculum tali (Fig. 3b). The third screw (screw C) was drilled from the calcaneal tuberosity to the anterior tubercle of the calcaneus (Fig. 3c). (3) The sequence of screw fixation: screw A was placed first because it was located in the medial load-bearing column of the calcaneus. After fixation of this screw, the height and varus/valgus alignment of the calcaneus could be well maintained. Then, screw B was used to fix the articular surface fracture fragment. Finally, screw C was used to maintain the length of the calcaneum. (4) The selection of the screw type and size: the 5.5 mm fully threaded cannulated screws were usually chosen as screws A and C, and lag screws should not be selected to avoid the loss of reduction. The 4.3 mm cannulated screw was usually chosen as screw B.

### Complications analysis

Wound complications is the most common complication in the operative treatment of the calcaneal fractures, especially those who underwent ORIF through an extended lateral approach, with an incidence of 27–33%[10]. This is especially true in patients with poor blood glucose control, peripheral vascular diseases, long-term smoking and severe soft tissue injury. In the present study, the incidence of wound complications was 16.7% in the ORIF Group, which is much higher than that in Group A (0%). The reason was mainly related to the different interference degrees of the two technologies on the soft tissue around the calcaneus. For ORIF with an extended lateral approach, extensive soft tissue dissection would lead to the destruction of the blood supply of the fracture block, the formation of potential dead space, injury of the lateral calcaneal branch of the peroneal artery, which was the main supply vessel covering the flap, and the extension of operative time [21, 22], and therefore incision complications were increased. However, with the application of minimally invasive percutaneous treatment by ligamentotaxis, reduction and fixation were both percutaneous, there was little interferences to soft tissue; thus, the incidence of incision complications was lower.

In the present study, one case in Group A (all types: 1/21, Sanders type IV: 1/2) and one case in Group B (all types: 1/18, Sanders type IV: 1/3) developed traumatic arthritis of the subtalar joint, all of which manifested as pain in the tarsal sinus during walking, especially on uneven roads. After oral administration of NSAIDs, the symptoms could be significantly relieved, and thus there were no further surgical treatments. Through imaging analysis, we found that these two patients both were Sanders type IV DIACFs. Postoperative CT indicated that the stepped displacement of the posterior subtalar articular surface were > 3 mm, which was similar to that reported by Tomesen et al. [14]. Among 39 patients with DIACFs who underwent percutaneous minimally invasive surgery, two patients who finally underwent subtalar joint fusion due to traumatic arthritis were both Sanders type IV DIACFs, and the stepped displacement of posterior subtalar articular after operation were > 2 mm. This finding indicated that the occurrence of traumatic arthritis was closely related to whether the reduction of the articular surface was satisfactory and that for severe articular surface comminuted fractures, especially for Sanders type IV DIACFs, the use of a minimally invasive percutaneous reduction technique should be done with caution because of its higher risk of traumatic arthritis.

## Limitations

The limitations of this study were as follows. First, this study is a single-centre retrospective study. Second, although the control group was established, the sample size was small, both in the study group and in the control group. Third, the follow-up time, which is a medium- and short-term follow-up, is not long enough. It is unclear whether the radiologic parameters of the calcaneus will undergo significant changes with a longer follow-up period, as described by Wang and Wei [23], who found that the Böhler angle had decreased at a mean follow-up of 40.4 months compared with the values obtained immediately after surgery.

## Conclusions

The use of minimally invasive percutaneous treatment by ligamentotaxis for Essex-Lopresti joint depression-type DIACFs can achieve similar clinical efficacy to traditional open reduction and internal plate fixation. Furthermore, it has the advantages of a shorter preoperative waiting time, hospital stay and a lower incidence of incision complications.

## Data Availability

The datasets supporting the conclusions of this article are included within the article.

## Abbreviations

AOFAS: American Orthopaedic Foot and Ankle Society
CT: Computed tomography
DIACFs: Displaced intra-articular calcaneal fractures
MFS: Maryland Foot Score
ORIF: Open reduction and internal fixation
SRJSR: Satisfactory rate of joint surface reduction
TCDR: Tri-plane calcaneal distraction reductor
VAS: Visual analogue scale
